# Predictors of Nursing Home Covid-19 Cases: a Community Vulnerability Approach

**DOI:** 10.1093/geroni/igab046.3635

**Published:** 2021-12-17

**Authors:** Matthew Peterson, Larry Lawhorne

**Affiliations:** 1 UNC Wilmington, Wilmington, North Carolina, United States; 2 Wright State University, Fairborn, Ohio, United States

## Abstract

It is well known that the Covid-19 pandemic has placed considerable burden on nursing homes, including from resident, facility, and community perspectives, among others. This study examined facility and community factors that were related to incident Covid-19 cases in nursing home facilities. N=12,473 US nursing homes were included in this study. Data from June 2020 - January 2021 from several publicly available sources were combined to create a dataset that included facility name, size, ownership, mortality rate, Covid case rate, personal protective equipment (PPE) and staff shortages, % white residents, and % Medicaid residents. Community factors included core-based statistical area (CBSA) Covid case rates, urban/rural, CBSA death rates, and the CDC’s Social Vulnerability Index (SVI). Zero-inflated Poisson regression models were used to determine predictors of 8-month Covid case counts, normalized by facility size. Results indicated that higher staff shortages, poorer facility rating, for-profit ownership, proportionally more Medicaid and non-white residents were all significantly associated with higher Covid case rates over 8 months (all P < 0.0001). Significant community level predictors of higher cases included urban setting and higher SVI. PPE shortages was not associated with higher case counts. Of all the factors included, SVI was the strongest predictor of Covid case counts. This large US study assists in determining critical facility and community factors that predict increasing Covid burden in nursing homes. Particularly, SVI is an important factor in determining facility and public health policy, and targeting resources in large scale health crises such as the Covid-19 pandemic.

